# A Meta-Analysis of Different Types of Cardiac Adipose Tissue in HIV Patients

**DOI:** 10.1155/2020/8234618

**Published:** 2020-12-09

**Authors:** Guang Song, Wei Qiao, Lu Sun, Xiaona Yu

**Affiliations:** Department of Ultrasound, Shengjing Hospital of China Medical University, Shenyang, China

## Abstract

**Background:**

Antiretroviral therapy transformed HIV infection into a chronic disease but accelerated cardiovascular disease (CVD). Both of epicardial adipose tissue (EAT) and pericardial fat (PCF) have close relationships with CVD. The associations between these two cardiac adipose tissue and HIV are unclear.

**Methods:**

Eligible studies were searched in PubMed, Embase, Web of Science, and Scopus from database inception to March 24, 2020. The summarized standard mean difference (SMD) or weighted mean difference (WMD) with 95% confidence intervals (CIs) was used to assess the association between EAT/PCF and HIV. Subgroup analysis was performed based on EAT types. Trial sequential analysis was conducted to estimate whether the evidence of the results is sufficient.

**Results:**

In total, 2561 HIV patients and 1767 non-HIV participants were included. Compared to the control group, EAT was significantly higher in the HIV overall group and subgroup with EAT thickness (SMD = 0.59, 95% CI: 0.24–0.95, *P* = 0.001; SMD = 1.10, 95% CI: 0.41–1.79, *P* = 0.002); however, the EAT volume and PCF volume were unchanged in the HIV group (SMD = 0.16, 95% CI: -0.07–0.39, *P* = 0.169; WMD = 10.78, 95% CI: -14.11–35.67, *P* = 0.396). Trial sequential analysis indicated that the available samples were sufficient in the HIV overall group and subgroup with EAT thickness, and more studies are needed for EAT volume and PCF volume.

**Conclusions:**

EAT thickness was significantly higher in patients with HIV. The association between EAT/PCF volume and HIV needs more studies to confirm.

## 1. Introduction

Human immunodeficiency virus (HIV) is one of the most serious public health challenges in the world. By the end of 2018, 37.9 million individuals were infected with HIV globally, while around 770,000 people died of HIV-related illnesses worldwide in 2018 [1]. Individuals infected with HIV have an increased risk of cardiovascular disease (CVD) due to the chronic inflammation, aberrant immune activation, toxicity due to antiretroviral therapy (ART), and viral effects of HIV [2, 3]. A previous study predicted that 78% of individuals living with HIV infection will have CVD by the year 2030 [4]. Meanwhile, patients with HIV on ART are also associated with an increased risk of metabolic syndrome (MS) and obesity [5, 6].

Epicardial adipose tissue (EAT) is defined as the adipose tissue adjacent to the epicardium surrounding the heart, located inside the pericardial sac; pericardial adipose tissue, also named pericardial fat (PCF), delineated by the pericardial sac, is a local fat depot that surrounds the heart in which the coronary arteries are embedded [7, 8]. EAT and PCF are visceral heart adipose and have recently emerged as ectopic depots of interest to clinicians. Both EAT and PCF have close relationships with CVD [9, 10], MS [11, 12], and obesity [13].

There were several cross-sectional studies focused on the association between EAT/PCF and CVD risk in HIV patients [14, 15]. They found that EAT and PCF were associated with cardiovascular risk in those patients. On the other hand, studies were performed to assess EAT and PCF between HIV patients and non-HIV individuals [16, 17]. However, the results were inconsistent, and small sample sizes can affect the strength of the previous evidence. So, we conducted a meta-analysis of studies with the aim of providing a more comprehensive summary of currently available research to evaluate the association between these two cardiac adipose tissues and HIV.

## 2. Methods

### 2.1. Search Strategy

Two independent investigators independently searched PubMed, Embase, Web of Science, and Scopus from database inception to March 24, 2020, to identify the relevant studies. The following search keywords included “HIV”, “Human Immunodeficiency Virus”, “AIDS Virus”, “epicardial fat”, “epicardial adipose tissue”, “pericardial adipose tissue”, and “pericardial fat”. This study only focused on human studies. We conducted this meta-analysis using a predetermined protocol in accordance with the Preferred Reporting Items for Systematic Reviews and Meta-Analyses (PRISMA) reporting guidelines [18]. We followed the methods of Song et al. about “data extraction and statistical analysis” [19].

### 2.2. Study Selection and Exclusion

Original studies were eligible if the following criteria were met: (i) HIV patients as a case group and non-HIV individuals as a control group and (ii) quantitative measurement of EAT or PCF (volume or thickness) by echocardiography, magnetic resonance imaging, or computerized tomography. Original studies were ineligible if the following criteria existed: (i) reviews, letters, or case reports; (ii) having no non-HIV control group; (iii) invalid analysis; or (iv) not reporting the data necessary for calculating the mean and standard deviation of EAT or PCF. If there were several publications from the same study, the study with the most cases and relevant information was included.

### 2.3. Data Extraction and Quality Assessment

Data extraction was performed independently by two of the reviewers. Disagreements were discussed and resolved by consensus or by involving a third reviewer for adjudication. The extracted data included the first author, year of publication, country, case number, gender, mean age, duration since HIV diagnosis, ART percentage, duration of ART, current CD4^+^ count, total cholesterol, triglycerides, high-density lipoprotein cholesterol, low-density lipoprotein cholesterol, fasting glucose, body mass index (BMI), and measure of EAT and PCF. We used the Newcastle-Ottawa Scale (NOS) to assess the quality of all studies included [20]. The NOS contains eight items, categorized into three groups including selection, comparability, and exposure [20]. Stars awarded for each quality item serve as a quick visual assessment. Stars are awarded such that the highest quality studies are awarded up to nine stars.

### 2.4. Statistical Analysis

The pooled effects are presented as the standard mean difference (SMD) or weighted mean difference (WMD) with 95% confidence intervals (CIs). Heterogeneity was assessed using the *I*^2^ statistic. If there was no heterogeneity (*P* > 0.1 or *I*^2^ < 50%), a fixed effects model was used to estimate the pooled WMD; otherwise, a random effects model was utilized [21]. We conducted a subgroup analysis based on EAT types (“EAT thickness” and “EAT volume”). Sensitivity analyses were directed to assess the influence of the individual study on the overall estimate by sequentially removing each study. We used Begg's and Egger's tests to evaluate publication bias in the included studies. A *P* value < 0.05 was considered statistically significant for asymmetry. Statistical analyses were performed using Stata (version 14.0; StataCorp, College Station, TX, USA).

### 2.5. Trial Sequential Analysis (TSA)

The TSA was conducted to maintain a 95% confidence interval, a 20% relative risk reduction, and an overall 5% risk of a type I error and 20% of the type II error (a power of 80%). When the cumulative *Z*-curve crossed the trial sequential monitoring boundary or exceeded the required information size line, a piece of firm evidence might have been reached and no further studies are needed. Otherwise, additional studies are needed to continue until getting adequate required information size [22]. TSA (version 0.9.5.10, http://www.ctu.dk/tsa/) was used in the current study.

## 3. Results

### 3.1. Description of the Included Studies

**Figure 1:**
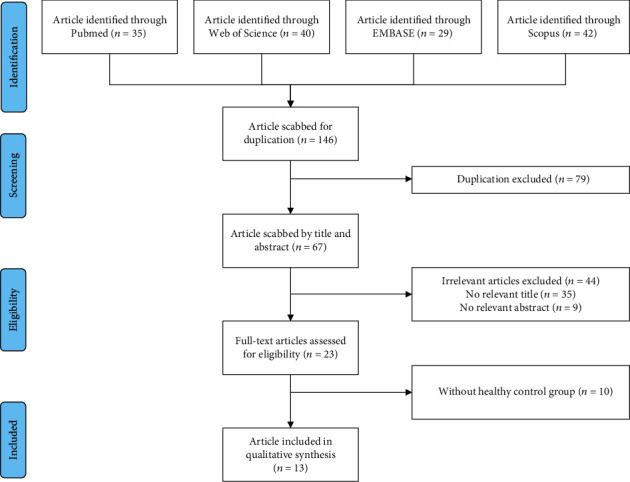
Flowchart of the study selection.

We identified and reviewed 146 potentially relevant publications from PubMed, Embase, Web of Science, and Scopus. After the application of the inclusion and exclusion criteria, thirteen observational studies were identified (Figure 1) [16, 17, 23–33].

The baseline characteristics of the involved studies are shown in Table 1. These studies were published between 2008 and 2020 in North America and Europe. Three studies measured the EAT thickness, five studies measured the EAT volume, and six studies measure the PCF volume. No study measured the PCF thickness. In total, 4328 participants (2561 HIV patients and 1767 non-HIV participants in the control group) were included. All NOS scores of the involved studies are more than six (details are shown in the supplement file (available here)).

### 3.2. Comparison of EAT between Patients with HIV and Controls

**Figure 2:**
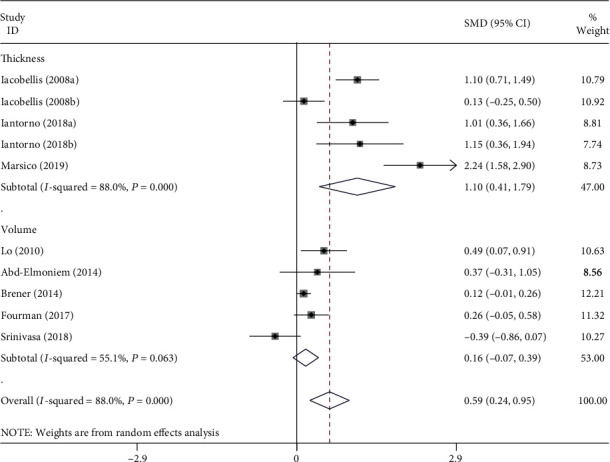
Subgroup analysis for the association between epicardial adipose tissue and HIV.

The EAT was significantly higher in the HIV group compared to the non-HIV group (SMD = 0.59, 95% CI: 0.24–0.95, *P* = 0.001). The subgroup analysis indicated that the EAT thickness was significantly higher in the HIV group compared to the non-HIV group (SMD = 1.10, 95% CI: 0.41–1.79, *P* = 0.002). However, the EAT volume was unchanged in the HIV group compared to the non-HIV group (SMD = 0.16, 95% CI: -0.07–0.39, *P* = 0.169) (Figure 2). Sensitivity analyses revealed that no apparent change occurred when an individual study was omitted. No publication bias was detected by Begg's (*P* = 0.152) and Egger's tests (*P* = 0.073).

### 3.3. Comparison of PCF between Patients with HIV and Controls

**Figure 3:**
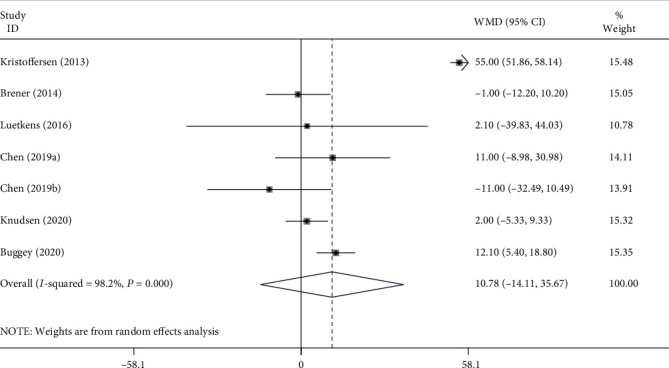
Forest plot for the association between pericardial fat and HIV.

The PCF volume was unchanged in the HIV group compared to the non-HIV group (WMD = 10.78, 95% CI: -14.11–35.67, *P* = 0.396) (Figure 3). Sensitivity analyses revealed that no apparent change occurred when an individual study was omitted. No publication bias was detected by Begg's (*P* = 0.548) and Egger's tests (*P* = 0.068).

### 3.4. Trial Sequential Analysis

**Figure 4:**
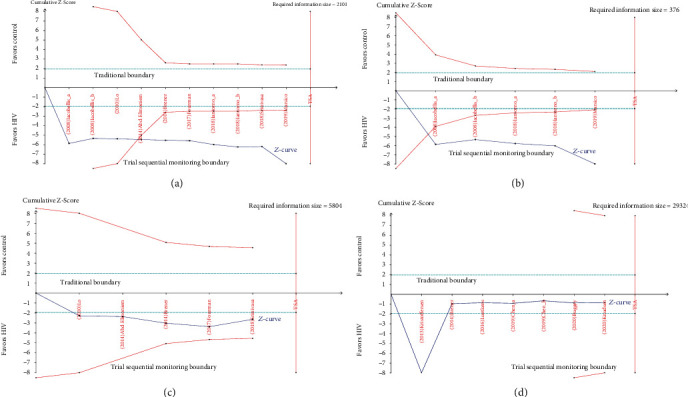
The results of trial sequential analysis: (a) epicardial adipose tissue overall group; (b) subgroup with epicardial adipose tissue thickness; (c) subgroup with epicardial adipose tissue volume; (d) subgroup with pericardial fat volume.

TSA revealed that the cumulative *Z*-curve passed the trial sequential monitoring boundary, suggesting sufficient evidence for a firm conclusion in the overall group of EAT and subgroup with EAT thickness; however, the cumulative *Z*-curve of EAT volume and PCF volume did not cross the trial sequential monitoring boundary or the required information size line, indicating that more studies are needed (Figure 4).

## 4. Discussion

This is the first meta-analysis that includes more than four thousand participants to assess the association between EAT/PCF and HIV. We found that EAT, especially EAT thickness, was higher in the HIV group compared to the non-HIV group. Furthermore, TSA indicated that the available samples were sufficient and firm evidence was reached; the sensitivity analysis further confirmed these findings. However, EAT volume and PCF volume were not associated with HIV, which needs more studies to support these null associations.

As the access to ART has improved throughout the world, the natural history of HIV infection has dramatically changed from a deathly disease to a chronic condition. ART helped people with HIV survival, but accelerated CVD is now an important cause of significant disability and premature mortality [34]. Some risk factors may be involved in the pathogenesis of CVD in HIV patients, including traditional risk factors, infection-related factors, and therapy-related factors. Epidemiological studies have shown an increased risk of about 50%–100% for CVD associated with HIV infection even after controlling for traditional risk factors [35].

Finding reliable markers to assess the CVD risk in HIV patients has always been the direction of clinicians, such as summarizing immune markers for CVD [35]. Recently, Buggey and Longenecker published a review paper and proposed a hypothesis that cardiac adipose tissue may serve as an important imaging marker of risk but may also directly mediate coronary artery disease and cardiac dysfunction [36]. EAT and PCF are the cardiac fat depots most concerned about. They are the independent predictors of CVD in the general population, including coronary artery disease and arteriosclerosis [37, 38]. The EAT and PCF can be accurately measured by echocardiography, magnetic resonance imaging, and computerized tomography [39, 40]. In our result, EAT thickness is the only index that has proven to be statistically significantly higher in HIV patients. EAT thickness has the potential to be a risk marker of CVD in HIV patients.

EAT is a small but very biologically active ectopic fat depot in direct contact with the myocardium and coronary arteries. EAT thickness not only is a simple and reliable marker of visceral adiposity in the general population [41] but also has the potential in HIV patients [42]. Obesity and visceral adiposity are well-known risk factors for CVD in HIV patients [43]. In our thirteen involved studies, EAT usually showed similar test efficiency with visceral adipose tissue measurement, better than BMI [23, 27] to assess obesity and visceral adiposity. Meanwhile, EAT is associated with CVD risk in HIV patients, independent of traditional CVD risk factors [44]. The clinicians could measure EAT to identify the phenotype of adiposopathy (i.e., “sick fat”) among treated patients with HIV infection; then, targeting these patients with lifestyle interventions and metabolic therapies may reduce the levels of chronic inflammation and immune activation that are thought to contribute to a wide range of HIV chronic comorbidities such as CVD [30].

The mechanism of EAT thickening in HIV patients is not totally clear. Based on the results of existing research, we propose the following: (I) 22% of individuals with normal BMI became overweight, and 18% of overweight individuals became obese within 3 years of starting ART [6]. Also, ART predisposes to ectopic fat deposition, including EAT [45]. (II) Many studies have demonstrated that chronic inflammation manifested as higher levels of inflammatory mediator, including IL- (interleukin-) 6, tumor necrosis factor receptor *α*-1, and C-reactive protein after ART treatment. Meanwhile, the increasing EAT usually was associated with those inflammatory mediators [46]. (III) The use of ART may cause serum lipid changes [47]. Dyslipidemia is the most prevalent concomitant cardiovascular risk factor in HIV patients [48, 49]. EAT was associated with high-density lipoprotein cholesterol and triglycerides [46].

## 5. Limitation

There were several limitations in this study. First, in the subgroup with EAT thickness, there were only three studies. We cannot use stratified analysis by an imaging method. Second, thirteen studies did not have very large sample sizes. We cannot investigate the effect of ethnicity on EAT or PCF. Third, the duration of ART could affect the EAT, which was not considered in this study due to the lack of the individual participant data [36].

## 6. Conclusion

EAT thickness was significantly higher in patients with HIV. The association between EAT/PCF volume and HIV needs more studies to confirm.

## Figures and Tables

**Table 1:** Baseline of the involved studies.

Study	Year	Country	Arms	*N*	Gender (male/female)	Mean age (years)	BMI (kg/m^2^)	Measure result	Imaging modalities	NOS score
EAT										
Iacobellis	2008	Canada	HIV_1	57	45/12	44.0	35.0 ± 2.5	7.1 ± 1.0 mm	Echocardiography	8
			HIV_2	52	42/10	44.5	27.0 ± 3.5	6.3 ± 2.0 mm		
			Control	57	40/17	48.4	35.0 ± 4.0	6.0 ± 2.0 mm		
Lo	2010	USA	HIV	78	78/0	46.5	26.1 ± 4.3	112 ± 54 mm^3^	CT	8
			Control	32	32/0	45.4	26.9 ± 5.2	85 ± 57 mm^3^		
Abd-Elmoniem	2014	USA	HIV	35	19/16	22	23.9 ± 5.7	27.5 ± 17.0 mm^3^	CT	7
			Control	11	3/8	25	26.1 ± 4.8	21.9 ± 3.0 mm^3^		
Brener	2014	USA	HIV	579	579/0	53.4	26.1 ± 4.4	121 ± 60 mm^3^	CT	9
			Control	353	353/0	55.9	27.5 ± 4.9	114 ± 52 mm^3^		
Fourman	2017	USA	HIV	121	80/41	46.7	28.0 ± 5.2	93 ± 55 mm^3^	CT	8
			Control	57	36/21	45.2	27.3 ± 4.8	79 ± 49 mm^3^		
Srinivasa	2018	USA	HIV	55	0/55	47.0	28.0 ± 1.0	54 ± 28 mm^3^	CT	8
			Control	27	0/27	47.0	29.0 ± 1.0	65 ± 27 mm^3^		
Iantorno	2018	USA	HIV_1	36	24/12	53.0	27.0 ± 4.0	16.3 ± 6.0 mm	MRI	7
			HIV_2	15	11/4	57.0	26.0 ± 8.0	13.9 ± 3.1 mm		
			Control	14	5/7	50.0	25.0 ± 3.0	10.7 ± 3.3 mm		
Marsico	2019	Italy	HIV	29	13/16	13	20.0 ± 4.0	3.16 ± 1.05 mm	Echocardiography	9
			Control	29	13/16	13.6	19.0 ± 6.0	1.24 ± 0.61 mm		
PCF										
Kristoffersen	2013	Denmark	HIV	105	93/12	47.4	24.7 ± 0.33	211 ± 13 mm^3^	CT	8
			Control	105	93/12	47.4	25.7 ± 0.35	156 ± 10 mm^3^		
Brener	2014	USA	HIV	579	579/0	53.4	26.1 ± 4.4	125 ± 84 mm^3^	CT	9
			Control	353	353/0	55.9	27.5 ± 4.9	126 ± 85 mm^3^		
Luetkens	2016	Germany	HIV	28	25/3	49.0	25.0 ± 4.0	140.9 ± 51.6 mm^3^	MRI	9
			Control	22	15/7	45.4	25.4 ± 2.9	138.8 ± 89.3 mm^3^		
Chen	2019	USA	HIV_1	67	67/0	53.0	27.±5.6	84 ± 44 mm^3^	CT	7
			HIV_2	38	0/38	52.0	34.0 ± 9.7	87 ± 40 mm^3^		
			Control_1	12	12/0	52.0	29.0 ± 4.9	73 ± 30 mm^3^		
			Control_2	8	0/8	46.0	38.0 ± 7.8	92 ± 25 mm^3^		
Knudsen	2020	Denmark	HIV	587	518/69	52	24.8 ± 3.5	192 ± 62 mm^3^	CT	9
			Control	587	518/69	52	26.6 ± 3.5	190 ± 66 mm^3^		
Buggey	2020	USA	HIV	100	38/62	54.5	27.4 ± 6.4	61 ± 25.9 mm^3^	CT	9
			Control	100	38/62	55.0	30.4 ± 5.6	48.9 ± 22.3 mm^3^		

BMI: body mass index; CT: computerized tomography; EAT: epicardial adipose tissue; MRI: magnetic resonance imaging; NOS: Newcastle-Ottawa Scale; PCF: pericardial fat.

## Data Availability

The data used to support the findings of this study are available from the corresponding author upon request.

## Supplementary Materials

Supplementary Materials Supplemental Table 1: summary NOS of the studies included in the meta-analysis. Supplemental Table 2: other clinical baseline of the involved studies.
