# Supplementary material for: A Meta-Analysis of Different Types of Cardiac Adipose Tissue in HIV Patients
Source: Biomed Res Int. 2020 Dec 9;2020:8234618. doi: 10.1155/2020/8234618 (PMC7746457; doi:10.1155/2020/8234618)
Supplement: Supplementary Materials — Supplemental Table 1: summary NOS of the studies included in the meta-analysis. Supplemental Table 2: other clinical baseline of the involved studies. [file 8234618.f1.docx]

**Supplemental Material**

Supplemental TABLE 1 Summary NOS of the studies included in the meta-analysis

|  |  |  |  |  |  |  |  |  |  |
| --- | --- | --- | --- | --- | --- | --- | --- | --- | --- |
| Study | **Selection (4 stars)** | | | | **Comparability (2 stars)** | **Exposure (3 stars)** | | | **Overall** |
|  | Is the case definition adequate | Representativeness of the cases | Selection of Controls | Definition of Controls |  | Ascertainment of exposure | Same method of ascertainment for cases and controls | Non-Response rate |  |
| Iacobellis (2008) | ★ | ★ | ★ | ★ | ★★ | ★ | ★ |  | 8 |
| Lo (2010) | ★ | ★ | ★ | ★ | ★★ | ★ | ★ |  | 8 |
| Kristoffersen (2013) | ★ | ★ | ★ | ★ | ★★ | ★ | ★ |  | 8 |
| Abd-Elmoniem (2014) | ★ | ★ | ★ | ★ | ★★ |  | ★ |  | 7 |
| Brener (2014) | ★ | ★ | ★ | ★ | ★★ | ★ | ★ | ★ | 9 |
| Luetkens (2016) | ★ | ★ | ★ | ★ | ★★ | ★ | ★ | ★ | 9 |
| Fourman (2017) | ★ | ★ | ★ | ★ | ★★ | ★ | ★ |  | 8 |
| Srinivasa (2018) | ★ | ★ | ★ | ★ | ★★ | ★ | ★ |  | 8 |
| Iantorno (2018) | ★ | ★ | ★ | ★ | ★★ |  | ★ |  | 7 |
| Chen (2019) | ★ | ★ | ★ | ★ | ★★ |  | ★ |  | 7 |
| Marsico (2019) | ★ | ★ | ★ | ★ | ★★ | ★ | ★ | ★ | 9 |
| Knudsen (2020) | ★ | ★ | ★ | ★ | ★★ | ★ | ★ | ★ | 9 |
| Buggey (2020) | ★ | ★ | ★ | ★ | ★★ | ★ | ★ | ★ | 9 |

Supplemental TABLE 2: Other clinical baseline of the involved studies

| Study | Year | Country | Arms | N | Duration since HIV diagnosis (years) | ART (%) | Duration of ART (years) | Current CD4^+^ (cells/mm^3^) | TC (mg/dl) | HDL-C (mg/dl) | LDL-C (mg/dl) | TG (mg/dl) | Fasting glucose (mg/dl) |
| --- | --- | --- | --- | --- | --- | --- | --- | --- | --- | --- | --- | --- | --- |
| **EAT** |  |  |  |  |  |  |  |  |  |  |  |  |  |
| Iacobellis | 2008 | Canada | HIV_1 | 57 | NR | 100% | 3.2 | NR | 209±18 | 37±4 | 139±10 | 161±10 | 109±3 |
|  |  |  | HIV_2 | 52 | NR | NR | NR | NR | 185±25 | 53±6 | 107±10 | 135±8 | 95±5 |
|  |  |  | Control | 57 | - | - | - | - | 201±15 | 40±5 | 131±10 | 152±10 | 100±8 |
| Lo | 2010 | USA | HIV | 78 | 13.5±6.1 | 95% | 7.1±4.6 | 523±282 | NR | NR | NR | NR | 94±11 |
|  |  |  | Control | 32 | - | - | - | - | NR | NR | NR | NR | 92±9 |
| Abd-Elmoniem | 2014 | USA | HIV | 35 | NR | 71% | 15±5 | 502±306 | 152±31 | 47±12 | 89±34 | NR | 91±26 |
|  |  |  | Control | 11 | - | - | - | 781±185 | 177±28 | 65±12 | 98±26 | NR | 86±5 |
| Brener | 2014 | USA | HIV | 579 | NR | 95.9% | 12.5±3.9 | 599±242 | 188±43 | 48±16 | 106±36 | 175±131 | 103±25 |
|  |  |  | Control | 353 | - | - | - | - | 192±36 | 53±16 | 112±32 | 128±78 | 102±32 |
| Fourman | 2017 | USA | HIV | 121 | 14.1±7.1 | 81% | 6.1±5.1 | 633±326 | NR | NR | NR | NR | NR |
|  |  |  | Control | 57 | - | - | - | - | NR | NR | NR | NR | NR |
| Srinivasa | 2018 | USA | HIV | 55 | 15.0±1.0 | 98% | 8.0±1.0 | 599±42 | 189±6 | 61±3 | 106±5 | 108±8 | 91±6 |
|  |  |  | Control | 27 | - | - | - | - | 184±5 | 60±3 | 102±5 | 110±13 | 83±3 |
| Iantorno | 2018 | USA | HIV_1 | 36 | NR | 94.4% | 1–15 | 611±480 | NR | 60±30 | 91±42 | 114±54 | NR |
|  |  |  | HIV_2 | 15 | NR | 73.3% | 1-15 | 619±254 | NR | 53±19 | 91±27 | 129±58 | NR |
|  |  |  | Control | 14 | - | - | - | - | NR | 55±40 | 120±70 | 88±50 | NR |
| Marsico | 2019 | Italy | HIV | 29 | 12 | 100% | 10.0±9.8 | 876±479 | 157±36 | 42±12 | 95±26 | 78±49 | 72±15 |
|  |  |  | Control | 29 | - | - | - | - | 156±46 | 54±21 | 101±29 | 74±52 | 82±28 |
| **PCF** |  |  |  |  |  |  |  |  |  |  |  |  |  |
| Kristoffersen | 2013 | Denmark | HIV | 105 | 12.3 | 8.9 | 8.9±0.4 | 636±25 | 225±4 | 52±2 | 139±4 | 191±16 | 101±1 |
|  |  |  | Control | 105 | - | - | - | - | 199±4 | 54±2 | 126±4 | 100±5 | 95±2 |
| Brener | 2014 | USA | HIV | 579 | NR | 95.9% | 12.5±3.9 | 599±242 | 188±43 | 48±16 | 106±36 | 175±131 | 103±25 |
|  |  |  | Control | 353 | - | - | - | - | 192±36 | 53±16 | 112±32 | 128±78 | 102±32 |
| Luetkens | 2016 | Germany | HIV | 28 | 9.7±6.9 | 39.3% | NR | 475±308 | 206±46 | 46±13 | 129±35 | 224±162 | NR |
|  |  |  | Control | 22 | - | - | - | - | 192±43 | 49±12 | 114±36 | 191±89 | NR |
| Chen | 2019 | USA | HIV_1 | 67 | NR | NR | >1 | 599±273 | 166±36 | 53±16 | NR | 138±91 | NR |
|  |  |  | HIV_2 | 38 | NR | NR | >1 | 662±498 | 192±37 | 59±20 | NR | 134±93 | NR |
|  |  |  | Control_1 | 12 | - | - | - | - | 165±35 | 54±15 | NR | 77±33 | NR |
|  |  |  | Control_2 | 8 | - | - | - | - | 176±23 | 49±10 | NR | 131±73 | NR |
| Knudsen | 2020 | Denmark | HIV | 587 | 15.4±8.9 | 98.6% | 11.9±6.6 | 708±282 | NR | NR | 112±38 | NR | NR |
|  |  |  | Control | 587 | - | - | - | - | NR | NR | 135±35 | NR | NR |
| Buggey | 2020 | USA | HIV | 100 | 11.7±2.2 | 97% | 11.1±2.4 | 531±199 | 217±56 | 58±16 | 135±44 | NR | NR |
|  |  |  | Control | 100 | - | - | - | - | 213±47 | 52±12 | 139±43 | NR | NR |

ART, antiretroviral therapy; BMI: body mass index; EAT, epicardial adipose tissue; HDL-C: high-density lipoprotein cholesterol; LDL-C: low-density lipoprotein cholesterol; NOS, Newcastle-Ottawa Scale; NR, no reported; PCF, pericardial fat; TC, Total cholesterol; TG, Triglycerides.
